# Promoting Mentalization in Clinical Psychology at Universities: A Linguistic Analysis of Student Accounts

**DOI:** 10.5964/ejop.v11i1.812

**Published:** 2015-02-27

**Authors:** Maria Francesca Freda, Giovanna Esposito, Teresa Quaranta

**Affiliations:** a Department of Humanities, University of Naples Federico II, Naples, Italy; b SInAPSi Center, University of Naples Federico II, Naples, Italy; c Psychologist; University of South Wales, Newport, United Kingdom

**Keywords:** account, mentalization, linguistic inquiry, practicum, university training

## Abstract

This study investigated the structure of mentalization (Bateman & Fonagy, 2012) in a training context. The dual purpose was to evaluate the effectiveness of practicum student training and whether the Linguistic Inquiry method (Pennebaker, 2000) could be used to evaluate the three dimensions of mentalization — relational, cognitive, and emotional. The training utilized the groups and their accounts as devices and mediators to conceptualize the relationship between self-mentalizing training, the academic context and the practicum experience. Accounts from 38 Italian students pursuing master degree in Clinical, Dynamic, and Community Psychology were analyzed by LIWC software. The Wilcoxon test showed a significant increase in mentalizing words during the middle and end of the term, as compared with the beginning. The results displayed a need to promote mentalization within academic settings and indicated the value of this competence for clinical psychology.

## Introduction

Recent studies on training and assessment of psychological competence within the well-known competence movement (Rodolfa et al., 2005) highlight a reflective feature of competence: a competent performance is only possible with reflective and critical behavior, which allows individuals to understand when, where, and how to arrange and move their own resources.

Pursuant with the international clinical psychology training debate (Spruill et al., 2004), we believe that reflective competence is not only a transverse competence, but that it is a core competence (Schweitzer et al., 2014). Consequently, we explored the types of formative devices that would be useful to promote reflective competences in the university setting. It is well known that this transversal university training-processes aims to encourage the development of reflective and methodological competences to teach reflective practice in clinical training (Burgess, Rhodes, & Wilson, 2013).

As recalled by Bion (1962), every path of knowledge represents an emotional undertaking, as it implies the concept of “being in relation with”, which is immersed in emotional and affective dynamics. If the trainees enact these emotional processes and are able to recognize and elaborate upon them with reflective thinking, then these dynamics can become a functional resource for the education of clinical psychologists. Studying to be a clinical psychologist necessitates learning skills for clinical assessment and intervention, as well as acquiring reflective competences. These reflective competences are an integral part of the university training relationship, in which a student will be able to effectively put these skills to use in future work scenarios.

The specificity of clinical psychology can be etymologically traced to the Greek word *klinè*, which describes the concept of relation, emotion, and reflective thinking on relationships (Freda, 2011; Salvatore & Freda, 2011). Therefore, we strongly believe that non-classroom experiences, like practicum (supervised practice), enable students to understand the emotional dimensions that course through relationships and allow them to confront the image of themselves as psychology students and their future identities and roles as psychologists (Hatcher & Lassiter, 2007; Laidlaw & Gillanders, 2011; Nel, Pezzolesi, & Stott, 2012; Reid, Dahlgren, Petocz, & Dahlgren, 2008).

It is within this context that the clinical competence of mentalization becomes a functional competence to train capable clinical psychologists.

In fact, mentalization is a reflective process that defines the behavior of individuals as the consequence of intentional mental states (Allen & Fonagy, 2006), thereby necessitating the ability to use different cognitive skills (e.g., reflection, interpretation, mirroring) to signify relationship affective dynamics. Nevertheless, mentalization shares a common aspect with the core of clinical psychology, namely, the significance of relationships and the need to develop reflective thinking regarding the emotions that arise from relationships.

Interest in mentalization to treat clinical pathologies is now soaring in psychotherapy settings (Bateman & Fonagy, 2012), yet on the other hand, interest is also deepening in normative settings, such as formative contexts.

Data from research studies conducted in different schools (Bragin & Bragin, 2010; Slade, 2007; Twemlow, Fonagy, & Sacco, 2005) has shown that the competence of mentalization is a supportive learning tool, because it serves as a scaffolding function in student learning (Freda, Esposito, Martino, & González-Monteagudo, 2014; Padykula & Horwitz, 2012). In contrast, there are only a few studies that investigate mentalizing competence in an academic context with a non-psychotherapeutic perspective.

According to Fonagy (Fonagy et al., 2009), the importance of mentalization is not just for the psychopathologic field; it can more broadly be applied to support individuals who are adapting to different community settings throughout life.

Our research intends to foster mentalization competence among university students through a Reflective Practicum Group (RPG). First, we describe the RPG and its principal devices, “account” and “group”. Next, we focus on how such devices can be considered media for the promotion of mentalization in the learning process. We finally discuss the Linguistic Inquiry method, which indicates an increase in mentalization competence.

### Research Context

For a master's degree in clinical, dynamic, and community psychology, practicum activities for students in the first and second year of university cover a total of 200 hours: 124 with different traineeship institutions that are affiliated with the university and specialized in offering different types of psychological services to users; 60 with individual work, and 16 in Reflection Practicum Groups (RPG). RPGs, which are carried out at the university, require attendance at eight sessions each lasting two hours, and are made up of 15-20 students and led by a clinical psychologist. RPGs halt productive action, related to both learning and participation, in praxis (Freda, 2008; Mälkki & Lindblom-Ylänne, 2012). The reflection group works as an “in-between*”* exploration of the relationship between universities and the institutions or services of psychological practice. In other words, it eases the transition between the theoretical models learned during the university experience and the psychological praxis carried out for the first time during the practicum. The RPG is a training device that allows students to analyze the anxieties connected with the crossing of a boundary line between the university and the world outside, between the role of student and that of a future clinical psychologist. The aim of the training is to allow students to constantly work on the relationship between emotional dynamics enacted within the RPG university group setting and the isomorphic dynamics enacted within the external experience in the practicum facilities.

During RPGs, students are asked to write up different types of accounts. According to Carli and Paniccia (2005), accounts should depict a description-interpretation of events deriving from each author’s interpretative criteria. Critical reflection of an account with the members of the working group enables recognition of certain criteria and the related emotional experiences. Each member of the group is asked to give three different kinds of accounts, or “practicum accounts”, that progressively extend the individual’s point of view during the practicum. First, a significant personal impact is recounted from the student’s service experience, second, the individual describes a meaningful event experienced during practicum, and the student finally is required to give a summary of the experience as a whole.

Moreover, at the end of each session a volunteer transcribes an account of the RPG session, the so-called “group accounts”, and the narrative input is always the same (“Write an account about the last RPG group session”). The subsequent session opens with reading and group discussion about this last type of account. The volunteer students who wrote the account may even become spokesmen (Kaes, 1976) for the emotional and representational dynamics elicited by the working group.

### Formative Devices to Promote Mentalization

The narrative device has often been used in psychotherapeutic and formative contexts. Narrative has been shown to simultaneously act as a promoter and an indicator of the efficacy of a mentalizing intervention (Allen & Fonagy, 2006), as it offers a wide range of input that is linked to facilitator creativity (Bateman & Fonagy, 2012). Therefore, mentalization has a circular and bidirectional relationship with narration, since it is a narrative form, itself, (oral or written). Individuals who constantly mentalize create stories about the mental states of themselves and of others (Freda, Esposito, Martino, & Valerio, 2014).

Pennebaker extensively researched the function of narration (Pennebaker, 2000; Pennebaker & Seagal, 1999) and concluded that text is also a physical product of the working interaction between the clinician and the patient (Martino, Freda, & Camera, 2013). As a collaborative effort, it represents a mentalizing process in which the clinician and the patient keep their own and each other’s mental state in mind (Bateman & Fonagy, 2012).

The RPG account is a type of narration that may offer valid support for the comprehension of emotional dynamics created in the *hic et nunc* of the formative and clinical relationship; furthermore, it also represents a device with which individuals can evaluate the development of their own mentalizing competence (Rizq & Target, 2010). Through an account, one can share and verbalize emotional experiences, thereby facilitating explicit mentalization (Allen & Fonagy, 2006), which turns into a group narrative. The more experiences that are subjected to a group mentalizing process, the richer and more complex these narratives become (Karterud, 2011).

Members of a group offer a plurality viewpoints and different representations of the same experience. For example, the facilitator becomes a creative-social-mirror (Fonagy & Target, 1997) who keeps in mind the mental states of others, represents them, and translates them into a comprehensive language. This suggests that the “other” exists, as it is remembered and because it exists in another’s mind (Fonagy & Target, 1997). At the same time, group members are a reflective mirror for themselves and for the other members, as one both narrates and is narrated by others (Freda, De Luca Picione, & Esposito, in press). An individual who is not considered in another’s mind cannot, then, develop personal mentalizing potential (Bateman & Fonagy, 2012; Karterud, 2011). As a consequence, the group reproduces and amplifies historical and evolutionary situations, in which mentalization is normally developed. In Karterud’s opinion (Karterud, 2011), “the group is a training arena for mentalization”. Thus, when a subject recounts a personal experience, associations with other members of the group are formed and the process of symbolization is activated.

In order to further the development of mentalization, it is necessary to use a process-oriented approach, wherein the training and relationship of group members is an object for reflection (Karterud, 2011). This is possible as transferred phenomena lead to the reenactment of emotional dynamics. “Mentalizing the transference” refers to the mentalizing process where participants reflect on the interactions that occur in the *hic et nunc* of the group setting and focalize on others’ minds during mentalizing interventions. Hence, it becomes possible to redefine one’s own perceptions through the way in which other group members, or the facilitator, apply meaning to the mental states of others (Karterud & Bateman, 2012).

Therefore, the group labels emotions, placing the aporetic (unrefined and vague) in basic emotional categories, which permits implicit mentalization and exploration to create meaning. The process becomes more complex and complete the more these emotions are reconsidered in relation to how they are enacted. This process will most likely lead to mentalized affectivity (Bateman & Fonagy, 2012), whereby individuals, in empathy with others, become conscious of their own emotions and begin to fully understand the meaning of the affective state during the mentalizing process (Allen & Fonagy, 2006).

Nevertheless, it is difficult to measure the progress of mentalization in the intervention experiences and there are different methods for its analysis (Esposito, Rainone, & De Luca Picione, 2014). The most important is represented in the Reflexive Function Scale (Fonagy & Target, 1997) and its revisions (Fonagy & Ghinai, 2008). Studies that measured specific dimensions have also been conducted. Though not exhaustive, it is worth mentioning the analysis on the construct of empathy and on the reading of non-verbal cues (Padykula & Horwitz, 2012) and more recent surveys on mentalizing tasks (Beaulieu-Pelletier, Bouchard, & Philippe, 2013). In this paper, we use Linguistic Inquiry of Language (Pennebaker, 2000) to measure mentalization development. This methodology has been widely tested in many contexts, with the purpose of demonstrating the usefulness of the narrative device in improving mental and physical health. Yet, we hypothesize that this method is also useful to analyze the development of mentalizing competence. According to Pennebaker, words reflect, but do not cause, mental states; they act as an epiphenomenon that speaks and informs about psychological and emotional processes. The lexical analysis of language thus enables the dynamic interpretation of mental and emotional processes and the ability to follow transformative and evolutionary changes, which are also reflected with language (Tausczik & Pennebaker, 2010).

### Goal

Our main objective was to analyze the effectiveness of the Reflective Practicum Group (RPG) in promoting mentalization. We analyzed the group accounts written by volunteer students through lexical analysis with LIWC 2007 (Linguistic Inquiry and Word Count) software. We hypothesized that the word analysis used in the accounts could provide information about the level and quality of the mentalization.

Another objective was to show whether and, if so, how the LIWC can be adjusted to be a useful method for the evaluation of mentalizing competence. In particular, we assume that lexical analysis highlights any changes concerning the three dimensions that constitute mentalization—cognitive, emotional, and relational (Bateman & Fonagy, 2012). These dimensions can be traced to specific lexical categories of the software.

## Method

The analysis was performed on the corpus of group accounts. The decision to analyze such accounts was threefold: 1) These accounts can be considered a kind of group narrative, in which every single group member is a spokesman (Kaes, 1976), 2) It is assumed that voluntary accounts are not perceived as compulsory tasks to be completed, 3) There was the same narrative input for every group account.

We analyzed a total of 38 accounts from six RPGs during the academic year 2008/2009^i^. Accounts were collectively analyzed and we identified different phases:

- Phase I contains the accounts from the first and second session, for a total of 13 accounts,
- Phase II includes accounts from the third, fourth, and fifth session for a total of 13 accounts,
- Phase III includes accounts from the sixth and seventh session for a total of 12 accounts.

This triphasic grouping reflects the three main periods characterizing the RPG term. Moreover, it mirrors the logic with which the “practicum accounts” were fulfilled.

In particular, the software counts the words in a text by comparing those recognized with its own internal dictionary. It then counts the percentage frequency that selected words appeared in the text (Pennebaker & Francis, 1996). The dictionary consists of six macro-categories, which in turn are divided into categories and subcategories^ii^.

### Procedure

The first step in our analysis was to expand the software dictionary to make it more functional for this specific context, since the LIWC had not previously been used for investigations in educational contexts, such as reflection groups.

Since the software does not provide specific categories for the structure dimensions of mentalization, the next step was the identification of LIWC categories and subcategories that could be useful in exploring mentalizing processes and could represent its cognitive, emotional, and relational dimensions. Therefore, within the macro-category of *Psychological Process*, we identified the following categories and subcategories that we then defined as "mentalizing" dimensions:

1. The ***Cognitive Processes*** refer to the cognitive and meta-cognitive dimension pertaining to mentalizing (e.g., “causes”, “knowledge”, “thought”). In particular, among the various sub-categories that constitute Cognitive Processes, we identified two, *Causation* and *Insight,* which are considered the most representative of the cognitive processes (Pennebaker & Francis, 1996). The first includes lexemes that refer to dimensions of understanding, explanation, and connection of mentalizing (e.g., “because,” “effect”, “hence”). The *Insight* subcategory refers to cognition (e.g., “think”, “know”, “consider”).
2. The ***Affective Processes*** more properly outline the emotional dimension of mentalization (e.g., “happy”, “sad”, “anger”), which is defined in the software through different sub-categories. Specifically, among others, we identified the positive-negative polarity via the subcategories *Positive Emotion* (e.g., “love”, “nice”, “sweet”) and *Negative Emotion* (e.g., “hurt”, “ugly”, “nasty”), which are considered the most exemplificative of the affective processes (Pennebaker & Francis, 1996).
3. The ***Social Processes*** define relational and intersubjective mentalization (e.g., “mate”, “together”, “share”).

The extension and adaptation of the LIWC dictionary was based on the same criteria adopted by the inventor Pennebaker (2000). Specifically, we ​​used an Italian dictionary to pinpoint the literal meaning of each word^iii^.

We then performed a lexical analysis of the 38 group accounts, divided them into three phases, and applied the Wilcoxon test, in order to analyze relevant changes over time. Moreover, we used some accounts extracts to exemplify data interpretation. For this reason, parts of these texts are displayed in the discussion section.

## Results

**Figure 1:**
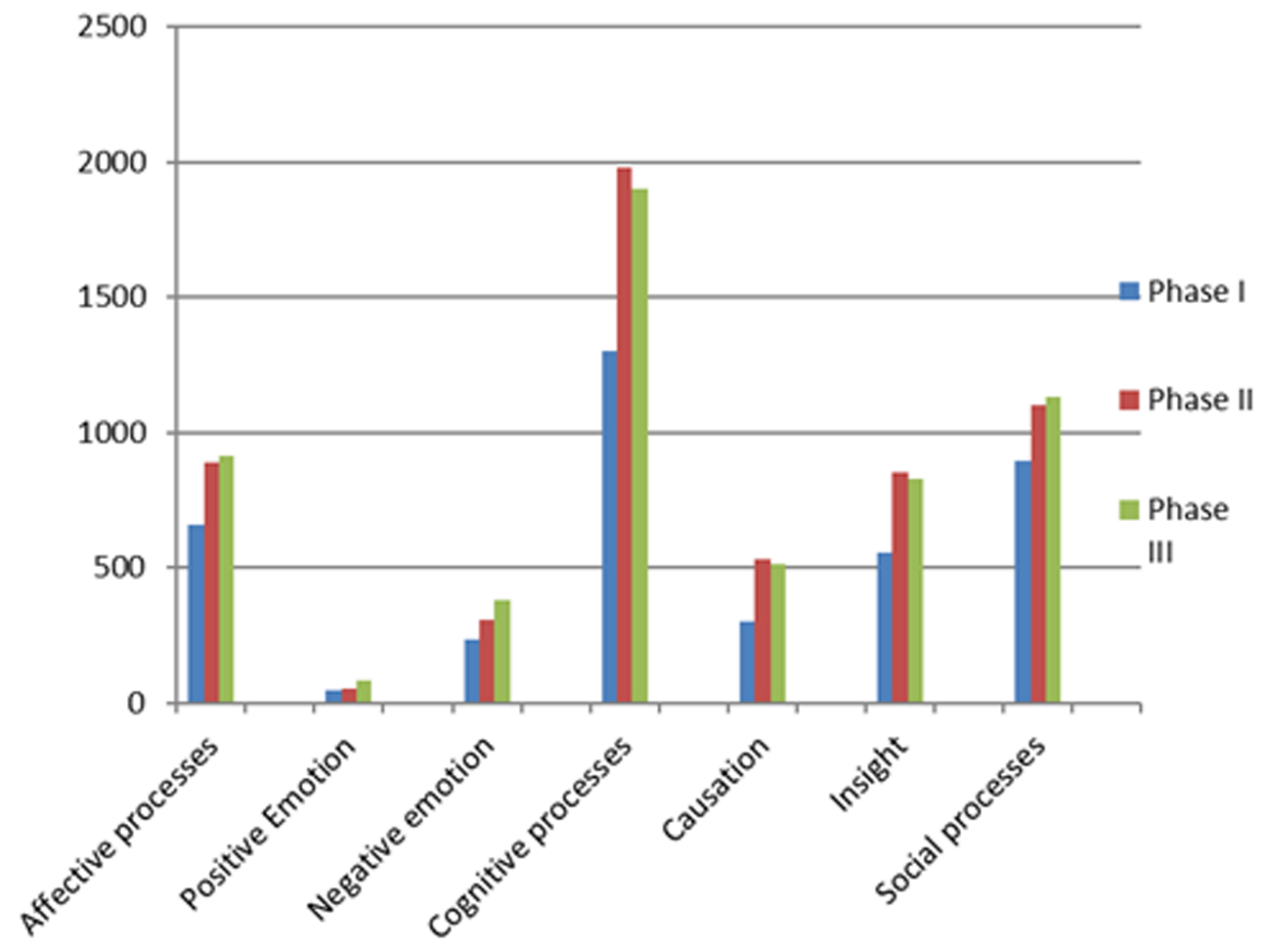
Number of words from mentalizing categories and subcategories in the three phases of RPGs.

In total, the accounts had 33.686 mentalizing words, which tended to increase in number in the transition between the phases (Phase I: 9.019; Phase II: 12.150; Phase III: 12.517). Figure 1 displays the change in the number of words for each category and subcategory and within each phase.

From Figure 1, we can see that, compared to the emotional and social categories, words from the Cognitive Processes category are the most frequent and show a high frequency of lexemes already in Phase I.

In addition, there is an increase of word numbers for each "mentalizing" category and subcategory from Phase I to Phase II, while, in the transition from Phase II to Phase III, there is a further increase in words in the Affective Processes category and its subcategories "Positive Emotion" and "Negative Emotion", as well as in the Social Processes category. In contrast, the Cognitive processes category and its subcategories "Causation" and “Insight" again show a decrease in the number of words in Phase III (without reaching the levels of Phase I).

Therefore, we applied the Wilcoxon test for multiple comparisons between pairs of phases to calculate the significance of such changes.

In the following three tables, we report the output of the Wilcoxon test for each of the three mentalizing dimensions (Table 1, 2, and 3).

**Table 1:** Wilcoxon Test for the Emotional Dimension of Mentalization

	Phase	Word Number	Mean Rank	Wilcoxon *Z*	Asymp. *p*
Affective Processes	I	658	14.08	-0.385	.700
	II	888	12.92		
	I	658	9.85	-1.999	**.046**
	III	914	15.64		
	II	888	9.69	-2.116	**.034**
	III	914	15.82		
Positive Emotion	I	45	11.09	-1.642	.101
	II	52	15.92		
	I	45	8.81	-2.809	**.005**
	III	82	16.86		
	II	52	9.42	-2.331	**.020**
	III	82	16.14		
Negative Emotion	I	236	14.08	-0.389	.697
	II	308	12.92		
	I	236	9.23	-2.468	**.014**
	III	382	16.36		
	II	308	8.54	-2.990	**.003**
	III	382	17.18		

*Note.* Boldface indicates significant results.

**Table 2:** Wilcoxon Test for the Cognitive Dimension of Mentalization

	Phase	Word Number	Mean Rank	Wilcoxon *Z*	Asymp. *p*
Cognitive Processes	I	1298	10.46	-2.026	**.043**
	II	1982	16.54		
	I	1298	12.69	-0.145	.885
	III	1902	12.27		
	II	1982	14.62	-1.543	.111
	III	1902	10.00		
Causation	I	299	11.46	-1.361	.174
	II	528	15.54		
	I	299	10.62	-1.421	.155
	III	514	14.73		
	II	528	12.54	-0.029	.977
	III	514	12.45		
Insight	I	554	12.42	-0.718	.472
	II	850	14.58		
	I	554	10.62	-1.421	.155
	III	826	14.73		
	II	850	11.96	-0.406	.685
	III	826	13.14		

*Note.* Boldface indicates significant results.

**Table 3:** Wilcoxon Test for the Relational Dimension of Mentalization

	Phase	Word Number	Mean Rank	Wilcoxon *Z*	Asymp. *p*
Social Processes	I	895	11.81	-1.130	.258
	II	1102	15.19		
	I	895	9.38	-2.348	**.019**
	III	1129	16.18		
	II	1102	9.20	-2.319	**.020**
	III	1129	16.14		

*Note.* Boldface indicates significant results.

Analyses show a significant increase in the number of emotional words from Phase I to Phase III, and from Phase II to Phase III. This shows that the final sessions are characterized by an increase in the emotional dimension of mentalization.

With regard to cognitive lexemes, there was a significant increase only in the transition from Phase I to Phase II for the Cognitive Processes category, while there were no significant changes in the lexemes belonging to subcategories "Causation" and "Insight". The cognitive dimension of mentalization, although not represented by subcategories in which mentalization has been operationalized, thus showed a greater increase in the core of the training sessions.

The same trend that was observed for the affective categories was also found for the Social Process category: the final sessions accounts are characterized by an increase in the relational dimension of mentalization.

## Discussion

In comparing descriptive data, it is most noticeable that Phase I had the lowest use of mentalizing words.

Moreover, by analyzing what happens internally during Phase I, it can be noted that, in comparison with the emotional and social categories, there was a large number of words related to the cognitive dimension of mentalizing (Cognitive Processes). The high frequency of cognitive words allows for the assumption that this dimension is more easily "narrable" for students already in the early sessions of the training experience. Instead, during Phase I, it is more complicated to narrate about the processes of social sharing within the interpersonal context of RPG and the practicum, and even more so to talk about emotions, especially the positive ones.

Below are a few examples extracted from accounts written during Phase I (first and second sessions). These excerpts show how many accounts still move according to a predominantly descriptive logic, without reference to mental states of an emotional or relational nature:

“University is a very ‘removed’ institution, it only teaches theoretical models, which are not linked with what happens in ‘real’ psychological practice” (First session).

“I don’t learn anything at practicum! I learned more if I remain at home and study for the exam!” (Second session).

The descriptive style is also evident from the use of the word “report”, instead of “account”:

“This report concerns the 1^st^ RPG session. On Monday the 19^th^, the RPG experience started and we defined goals and purposes of these sessions” (First session).

In the transition to Phase II the accounts began to be characterized by increasing numbers of mentalizing words that related to all the three dimensions in which the mentalization had been operationalized. Students narrated more about positive and negative emotions spent (in person or by others) during the RPG experience and practicum; they started to make more references to what they or other figures believed in relation to the experiences in which they were involved, and, finally, told more about sharing, comparison, and often conflicting processes, implemented within their own educational contexts.

Below are other examples from accounts of Phase II (third, fourth, and fifth sessions):

“What I notice, while I am writing this account, is that, like my colleagues, I feel strongly inadequate. I have felt the need to take the floor to describe my point of view” (Fourth session).

“Having thousands of doubts helps us to carry on and drives us to make meaning out of events with the purpose of always having a more complete vision of how we live. I believe that this dynamic should be a subject for reflection, in order to grasp the functional behavior of the practicum experience and the university” (Third session).

“I realized that it is easy to get angry with others: my tutor, [home] university, and my colleagues. I think that this anger could help me, but it will not solve my problems over time” (Fifth session).

Students, from the third, fourth, and fifth sessions, began to use the accounts as tools for reflection. The increasing and significant trend in cognitive words reflects a new skill in assuming different points of view within the same account and, in particular, the possibility of recognizing the active role of the “self-agent rule” in orienting their own actions in formative settings. We suggest that the significant increase in the use of cognitive and metacognitive words in Phase II expresses the achievement of a more analytical reading of the training process, a greater involvement, and a more active investment in the practicum experience. The soaring numbers of cognitive words in Phase II highlights the students’ ability to think about the "formative self" as an actor, rather than a spectator, of events (Freda, De Luca Picione, & Esposito, in press). This is equivalent to the assumption of an "I" position, or that of agency (Caston, 2011), in order to work on emotional dimensions. We believe that agency is an important aspect of mentalizing competence (Bateman & Fonagy, 2012), as it represents the ability to choose for oneself and take on responsibilities, taking into account possibilities and drawbacks of a situation (Caston, 2011).

Moreover, we speculate that the central sessions represent a sort of transitional area (Winnicott, 1971), a creative mental and relational space in which members of the group can engage in research and construction of meanings in the form of creative stories, in the form of an imaginative activity that allows for the creation of new meanings of experience (Bateman & Fonagy, 2012). The group, at this phase, becomes a buffer zone and a land of dual membership in which thoughts and feelings are played with as if they were real, and where reality can be deconstructed as if it were fantasy. We believe that gradually students began to experience the group as a relational and holding space (Winnicott, 1971), in which it became possible to share, exchange, and unburden issues.

In Phase III, there was a significant increase in the use of emotional and social words, as if, by the end, the RPG account would become the holding environment within which to amplify the emotional and social mentalizing processes. In particular, the significant increase of words in the sub-category "Negative Emotion" is interesting, as it is in our opinion, an indicator of the development of mentalizing.

In contrast to Pennebacker’s studies, we believe that the increase of lexemes in this category shows that students are learning to name and understand different mental states. In fact, the mentalizing competence involves awareness in recognizing and labeling both positive and negative life experiences. Sometimes they can be so painful that it seems impossible to give them a name and reflect on them at the beginning of a formative or therapeutic experience. However, labeling negative emotions can have a double function; it represents fruitless venting and, at the same time, causes an evolution in awareness and knowledge of an emotional state.

Nevertheless, the descriptive data reveal a slight difference between mentalized lexemes between Phases II and III.

The following examples display the co-presence of affective, cognitive, and social lexemes, which expresses the abilities of the trainees to connect the three mentalizing dimensions and link different poles of the formative experience:

“I believe that wondering aloud, exploring, and making sense of questions has spurred the reflective processes. What allowed this kind of process to come out is the account. L.'s account is an example of this reflective effort and a search of a meta-perspective. This kind of perspective lets us reflect on complex experiences like the practicum” (Sixth session).

“I realized that we all share a sense of uncertainty and a feeling of ‘being in-between’, which I think is one of the characteristics of a psychologist. Moreover, I believe that ‘being in-between’ is not a boundary, but, on the contrary, may become a resource” (Seventh session).

“I am realizing that frustration, anger and the sense of impotence are all linked to the apprenticeship and perhaps also to the idealization of the practicum experience and the career of professional psychologists” (Fifth session).

An analysis of the trends highlighted in Phase III led us to hypothesize that, by the end of the training experience, the RPG would have encouraged the development of Mentalizing Affectivity. Mentalizing Affectivity is characterized by a balance between thought and emotion; we believe that these emotional, cognitive, and relational dimensions are in a circular relationship to one another and evolve simultaneously.

The group learns to reflect and make new meaning of the feeling of “being in-between”. In fact, during this phase of their university career, students are in a middle point between being students/trainees and professional psychologists, between university contexts and the practicum. This frustrating and doubtful feeling of being on the “border” between groups is converted into a privileged position of knowledge; metaphorically, it is like being in a fjord where rivers converge and pass through the relationships that clinical psychology wishes to protect.

## Conclusion

Normative-formative contexts are innovative environments in which the mentalization theory could be applied. Nevertheless, we think that RPGs have many clinical characteristics and that university and practicum settings can be useful to promote mentalization. In light of this evidence, we assert that mentalization is not a psychopathologic construct, but a developmental one: it is a context-specific and relation-specific competence that can be promoted in each subject and in every context (Bateman & Fonagy, 2012). The caregiver (parent, psychotherapist, or facilitator), who plays the role of *keeping in mind the other's mind* (Fonagy & Target, 1997), and the relationship of this caregiver with the subject are the *condicio sine qua non* to promoting mentalization. This person must be trained to direct reflective processes about the “formative self” and the relationships that the students entertain exclusively within formative contexts, without going into other personal dimensions that should be treated in psychotherapeutic settings.

Unlike psychotherapeutic settings, RPGs occur only eight times. On the one hand, this may represent a limitation in building a complex competence like mentalization. On the other, however, it makes the RPG more “goal-oriented”, in part due to facilitating tools, such as the group itself and accounts. The group setting feeds the circular processes of meaning, wherein the individual takes and gives to the group in a bidirectional movement, or "me-us" (Karterud, 2011). The account, in its dual role as a methodological tool and narrative, amplifies these processes; it creates order in the narrative of the group and, at the same time, activates thought processes. We believe that the account can play the role of an Interpersonal Interpretative Function (IIF) (Allen & Fonagy, 2006), which allows students to mentalize the relationships that psychologists experience.

To return to the international debate on competence in clinical psychology, we posit that mentalization is one of the core competences that should be promoted within universities for the purposes of providing future professional psychologists with useful tools, with which they will be able to face complicated and unpredictable realities. In clinical practice, psychologists constantly have to mentalize and, according to Fonagy and colleagues (Allen & Fonagy, 2006), it is a tool for the psychologist, because it is only in the experience of one’s own humanity that scientific knowledge can be effectively applied in a clinical setting.

Our results lead us to conclude by highlighting both the limits of this study and related implications for the research on mentalization in academic settings.

Our study, although based on group and narrative devices, can only offer a hypothesis on how these devices can promote mentalizing competences. In order to overcome these limits, we could use auto- and hetero-evaluation tools in the future to measure the influential strength of devices on the efficacy of RPGs. Other methods for analysis, both qualitative and quantitative, could compare the results and then use data, so as to provide helpful feedback to students. For example, it would be interesting to also include the trainer’s assessments about trainees’ mentalization competences in future research, and to evaluate the effectiveness of RPGs, considering the role of facilitator as a variable. This is in response to the need to measure the weight of subjective variables that relate to the different expertise of the facilitator in mentalizing, identifying mentalizing processes in the *hic et nunc,* and in being able to support others in the acquisition of such a complex competence as mentalization.

One more limit is linked to the analytical strategy. In fact, though innovative, the lexical categories that were identified and chosen from among those of the LIWC’s dictionary may be not sufficient. For example, the Cognitive Processes category is composed of other sub-categories (e.g., Discrepancy, Tentative, Certainty, Inclusive, etc.) that may have contributed to the significant increase of the "Cognitive Processes" category in Phase II. This means that the choice of analyzing certain categories and not others is a complex methodological passage that affects the process of analysis of the three mentalizing dimensions. Specifically, future studies should take the complexity of the mentalizing processes of a cognitive nature into account, not only those related to the processes of causation or insight, but also those that refer to the ability to make inferences, to adopt conjunctive rather than disjunctive ways of thinking, or to highlight discrepancies and differences of opinion.

Moreover, it is necessary to integrate the lexical analysis with a qualitative-interpretative analysis of the accounts, in order to avoid losing the dynamics of developmental stages. We believe that a qualitative analysis is also needed to interpret an increase in the use of mentalizing words. In fact, the intense reference to mental states is not always a good index of effective mentalizing competence, but, as with certain psychopathologies, it can also be an expression of dysfunctional hypermentalizing. In this sense, future research should focus on the possibility of articulating the relationship between "word" and "context", that is, between the types of lexemes used and the processes that have made the textual expression possible.
